# Developing a Smoking Cessation Intervention for Low Income and Minority Women

**DOI:** 10.4172/2167-0420.1000309

**Published:** 2016-04-25

**Authors:** Nefertiti C duPont, Martin C Mahoney, Linda S Kahn, Bonnie M Vest, Christy A Widman, Nikia S Clark-Hargrave, Deborah O Erwin

**Affiliations:** 1 Department of Gynecologic Oncology, Roswell Park Cancer Institute, Buffalo, New York, USA; 2 Departments of Medicine and Health Behavior, Roswell Park Cancer Institute, Elm and Carlton Streets, Buffalo, New York, USA; 3 Department of Family Medicine, University at Buffalo, The State University of New York at Buffalo, Buffalo, New York, USA; 4 Division of Cancer Prevention and Control, Roswell Park Cancer Institute, Elm and Carlton Streets, Buffalo, New York, USA

**Keywords:** Tobacco cessation, Minority women, Low income women, Community based participatory research, Human papillomavirus, Cervical dysplasia

## Abstract

**Objective:**

The objective of this qualitative pilot study was to elicit patient and provider feedback on how to develop a smoking cessation program for low income women with cervical dysplasia in an urban Women’s Health Center.

**Methods:**

A community-based participatory research project incorporating a focus group and structured interviews was utilized to elicit feedback on how to develop a culturally appropriate smoking cessation program appealing to low-income and minority women smokers.

**Results:**

Qualitative data from 13 patients, 4 nurses, and 6 staff members collected between January 2012-August 2012 described the challenges of finding effective mechanisms for cessation interventions that met the schedules and needs of low income and minority patients. Input from office staff indicated insufficient educational resources to offer patients, limited skills to assist patients and the importance of perceived patient readiness to quit as barriers to creating an effective smoking cessation program.

**Conclusion:**

Smoking cessation services targeting low-income and minority female smokers can be enhanced by providing clinic staff with patient education materials and smoking cessation training.

## Introduction

Cervical cancer disproportionately affects low income women, frequently due to limited access to health care services which leads to a lack of pap test screening [1,2]. Persistent infection with the oncogenic types of human papillomavirus (HPV) causes cervical dysplasia and if left untreated can advance to invasive cervical cancer [3].

Minority and low socioeconomic communities are at a higher risk for developing cervical cancer due to limited access to health care services, and higher smoking prevalence which is associated with impaired clearance of HPV infection [4–7]. Smokers have higher rates of cervical dysplasia persistence than non-smokers and are at an increased risk for developing invasive cervical cancer [8,9]. Women frequently visit community health clinics for Pap test screening and women who have an abnormal result are often asked to return for a follow up visit. Repeat pap testing and cervical colposcopy offers a teachable moment when patients can receive additional instruction on how to treat and prevent cervical dysplasia, including stopping smoking. The objective of this qualitative pilot study was to elicit patient and provider feedback on how to develop a smoking cessation program for low income women with cervical dysplasia in an urban Women’s Health Center (WHC).

## Methods

A community-based participatory research pilot project was designed using individual semi-structured interviews and a focus group to inform the development of a culturally appropriate smoking cessation program targeting low-income female smokers at an urban WHC. Institutional review board approval was obtained prior to study initiation. All participants gave written informed consent prior to inclusion in the study.

### The setting

Buffalo, New York has a population of 261,310 with African Americans comprising 39% of the citizens of the city [10]. Buffalo ranks among the poorest cities in the United States [10]. A partnership with an urban WHC in Buffalo was developed. The WHC provides 6,700 clinic visits per year including over 2,100 new patient visits. The clinic focuses on women’s reproductive health needs and performs pap tests and cervical colposcopy; the center performs over 1,500 pap tests annually and 18% of all pap tests are abnormal. Approximately 47% of patients seen at the WHC are African American and 42% are white; 98% of patients are of reproductive ages. The office staff of the WHC assists in all aspects of patient care and the nurses and nurse practitioners are responsible for the day-to-day health care needs of patients. The second clinical setting is a gynecologic oncology clinic also located in Buffalo, treating women with gynecologic malignancies and premalignant conditions. The gynecologic oncology center is a part of a National Cancer Institute designated Comprehensive Cancer Center and serves as a tertiary referral center for the WHC.

### Study design

The goal of this pilot study was to elicit gynecologic patient and WHC staff feedback on how to develop a smoking cessation program at the WHC. A formative research plan incorporating qualitative methods was implemented to elicit detailed feedback from patients and office staff on the potential design of a smoking cessation intervention, specifically linked to women at high risk for HPV.

The pilot study was designed to use a nonprobability, purposive sample [11] to include representative participants from two groups:

1. Young women who are smokers or former smokers with previous or current abnormal pap examinations or positive HPV status.
2. Clinical professional and clerical staff from the WHC who serve these young women. This sampling process for patients aimed to include at least 10 women or until responses reached “saturation” (responses repeating the same information); and for WHC staff, a majority were desired. Structured interviews and one focus group were undertaken to obtain participants’ input on preferred types of smoking cessation interventions. Analysis was based on a grounded theory approach in which the authors identified categories and concepts emerging from the participants’ responses. Themes and categories were compared among the study team to approach consensus [11].

All interested female patients 18 years and older from the WHC and the gynecologic oncology clinic that self-identified as either current or former smokers were eligible to participate in structured interviews. Recruitment relied upon promotional flyers placed in the WHC and by identifying patients who met the eligibility criteria during their scheduled gynecologic oncology clinic visit. The structured interviews asked patients (n=13) questions about their smoking history and barriers to smoking cessation, and about components of a successful smoking cessation program.

Nursing staff from the WHC (n=4) were also invited to participate in semi-structured interviews lasting approximately 30 minutes. The clerical and support staff at the WHC were also invited to participate in one focus group (n=6) to discuss the importance of smoking cessation in the WHC. The structured interviews and focus group asked the nurses and clinic staff questions about patient tobacco cessation counseling, treatment and education. The staff was also asked about resources and clinical tobacco cessation approaches they considered feasible and beneficial for the WHC patients. All participants were remunerated for their time with a $25 gift card from a local grocery store.

Interview transcripts were analyzed by three members of the research team using the content-driven immersion-crystallization approach to identify patterns and common themes [12].

## Results

We recruited a total of 23 participants (13 patients and 10 WHC staff). Six WHC office staff members participated in one focus group and four nurses from the WHC completed structured interviews. Multiple attempts were made to recruit patients from the WHC, however we were not successful in getting any to participate in a focus group or structured interview regardless of incentives. None of the patients were willing to spend any more time than their planned clinical appointment as they were negotiating health care during work or school breaks. Thirteen women recruited from the gynecologic oncology clinic completed structured interviews; eight were current smokers and five were former smokers, ages 18–73. Eleven of these patients had a history of an abnormal pap test. Patients at the gynecologic clinic were more willing to participate as they had greater clinic wait times and allowed more time for their visits than the WHC patients. Data collection was completed by August 2012.

## Patient responses

All patients interviewed (n=13) expressed a desire to stop smoking due to the health risks associated with tobacco use and several patients felt they could quit on their own in the future when they were ready. One recurring theme identified an association between smoking and alcohol use (Table 1). One patient stated:

…*when I was pregnant with my son I didn’t smoke for about a year and a half and then I started smoking again. I could casually drink again and that’s basically what I would do, drinking and smoking*.

When asked how a smoking cessation program should be designed several patients stated they wanted individualized information on smoking cessation (Table 2). Many patients (n=7) were interested in a social media based format for cessation but only if the information was not too intrusive in their everyday lives. When asked about the utility of text messaging to encourage smoking cessation one patient stated:

*For me personally, I probably would ignore them. I get a lot of messages like that from my phone company and I just delete them*.

However, another patient stated:

*Honestly, I think it depends on the person for me. Now that I know I have abnormal pap smears and that quitting smoking could help me, I think that I would enjoy it (text messages), but I think other people… because my boyfriend’s a smoker too, I think he would think it was really annoying. So, I think it depends on the person*.

Nine patients thought that Facebook would be a good medium to use for a smoking cessation program; however patients who were not current Facebook users and who stated they were not computer savvy were less enthusiastic about this approach. When a patient was asked about the dangers of tobacco use she stated:

*Putting dumb pictures of your lungs rotting and stuff doesn’t work. Now you’ve grossed me out and I want a cigarette*.

Despite this one comment most patients found the graphic, disease-oriented television advertisements from the New York State Department of Health helpful. Overall, most patients reported that a smoking cessation program should be individualized in order to be successful.

## Nurses and office staff responses [all quotes are nurses]

Four WHC nurses/nurse practitioners participated in structured interviews and six office staff participated in a focus group. An analysis of the transcripts revealed that the nurses felt ill equipped to provide smoking cessation counseling and had few readily available resources to provide to smokers (Table 1). One nurse stated:

*There’s not a specific standard of care…we’re supposed to encourage people to stop smoking. There’s not a specific way that we’re instructed how to do that, but it’s important as a clinician if we see a patient who smokes to, you know, encourage them to stop smoking but we don’t have anything specific*….

The most consistent theme was that the nurses and WHC staff felt that their patient population face social challenges and they worried that despite their efforts many patients would continue smoking. One nurse stated:

*Patients will say “I’ve got too much going on to think about quitting right now.” You know because a lot of times they’re coming to us if there’s a problem that’s a semi-crisis type of situation*.

When asked about how to create a successful smoking cessation program another nurse stated that patient readiness to quit was very important. She went on to say:

*Give them adequate information, but they have to be willing to be receptive to the information that’s given*.

The nurses and office staff reiterated similar themes regarding the difficulty of incorporating smoking cessation in their practice: lack of printed educational materials in the clinic to provide to patients, importance of patient readiness and staff feeling ill equipped to offer tobacco cessation treatment and counseling due to lack of education and expertise. In addition, the office staff focus group felt that while smoking cessation is important, the younger patient population is not always receptive to these efforts.

## Discussion/Conclusions

The purpose of the pilot study was to solicit input from clinical staff and patients to inform the development of a successful smoking cessation program that would benefit the WHC. The major theme offered by participants to improve smoking cessation efforts was the need to address patient stressors and coping mechanisms and to individualize cessation programs. While most health care providers believe smoking cessation is important, recent studies have noted that only a small minority provide guideline recommended cessation support to their patients [12]. Clinicians can be encouraged to provide cessation treatment and counseling by providing specialized training to address educational deficiencies. It is likely that dedicated office staff could help to more effectively address smoking cessation, even in busy practices. This approach is consistent with both the Patient Centered Medical Home designation for primary care offices in the United States (US) and the “care manager” nursing model used outside of the US. The women’s health office which was the focus of this study was under-resourced and was unable to assign dedicated staff to the sole task of health promotion and disease prevention and care coordination. Accordingly our efforts included attempts to encourage and educate office staff to engage smokers in discussions of cessation as part of routine medical care. And as patients were not inclined to take extra time for any other appointments, including cessation within their WHC visit would be most important.

Female smokers with a history of an abnormal pap test were initially identified as the target study population for this research project because of the relationship between HPV persistence and nicotine dependence. The rationale for focusing on this group of smokers was the potential opportunity for a teachable moment when patients are informed of an abnormal pap test result. Many times patients are asked to return to the clinic for a repeat pap test and/or treatment for cervical dysplasia. These office visits provide an opportunity for the health care clinician to address smoking cessation at a time when the patient may be the most receptive to additional education and counseling support.

Designing a smoking cessation program tailored to the needs of the target population was the initial goal of this project. The WHC treat a high volume of patients annually and provides well woman exams including pap test screening. Based on a brief assessment conducted when the project was introduced to the WHC staff, the nurses at the WHC were knowledgeable regarding the revised pap testing guidelines [13] and the association between smoking and HPV persistence. However, as noted in the structured interviews and focus group, the WHC staff members reported that they lacked the necessary tools to offer smoking cessation treatment (pharmacotherapy and/or counseling) and patient educational resources.

Data from the structured interviews with patients and nurses allowed us to understand some of the barriers associated with smoking cessation in low income and minority women. Patient barriers to smoking cessation suggest the need to address the social influences of smoking including alcohol use and psychosocial stressors, topics which may best be addressed in individual counseling rather than a group intervention. Barriers expressed from the nurse interviews and the office staff focus group suggests that cessation efforts should influence patients at a time when they are ready to quit, which can be addressed in part by using every office visit as an opportunity to advise cessation and offer assistance in quitting [14].

Several changes were implemented at the WHC based on this pilot project. First, the nursing staff was given evidence-based education on smoking cessation to enhance their understanding of the first line medications used to support smoking abstinence. The nurses were taught the importance of addressing tobacco use at each clinic visit and they were given basic cessation counseling tips. The nursing and clinic staff were also provided with written materials to use for patient counseling and referral information to assist patients who were ready to quit [15]. The WHC also expressed interest in becoming a tobacco free campus and are working to accomplish this goal. Lastly, the WHC is in the process of implementing an electronic health record that will record smoking status for each patient and document a patient’s readiness to quit.

The main limitations of this study were the relatively small sample size and participation bias. However, for a formative pilot study, focused on patients with specific experiences (smoking and abnormal paps) and clinical staff serving these patients in a community clinic, the 23 participants provided adequate saturation of the specialized topics of interest and are considered appropriate for nonprobability sampling [16]. Recruiting patients from the WHC proved to be more challenging than we anticipated and despite multiple approaches, techniques and incentives, we were not able to get patients recruited in a timely manner, therefore, recruitment was expanded to include patients from the gynecologic oncology clinic at the academic medical center and this is a limitation of this study. This inability to have WHC clients take time for interviews or focus groups demonstrates the time and scheduling challenges for setting up any type of group cessation program. Nonetheless, the use of qualitative data collection from patients at the gynecologic oncology center provided opportunities for engagement, elaboration and clarification of responses from a similar group of participants. These pilot findings support the need to more effectively address smoking cessation, especially among women with HPV related cervical abnormalities, through both systems changes and clinician education [14].

One key lesson learned from this pilot project was that the office staff tended to focus on health care issues with which they are comfortable (i.e., women’s health, pap screening, sexually transmitted infections) and tended to avoid topics, such as smoking cessation, which they viewed as being more challenging. As a result, rather than designing a new cessation program, we refocused efforts toward equipping the nursing and office staff with the knowledge and skills to effectively provide smoking cessation services to their patients during individual clinical visits, especially as they relate to managing HPV-related disease.

## Figures and Tables

**Table 1:** Opinions from study participants on barriers to smoking cessation by group.

Group	Themes (# participants who mentioned theme)	Illustrative Quotations	Participant characteristics
GYN center patients (n=13)	Lack of interest and motivation from patients (n=4)	I got the patches, but I didn’t wear them. I’d just rip the patch off and go smoke a cigarette	Smoker diagnosed with cervical dysplasia
	Lack of understanding of the long term health complications (n=4)	I wouldn’t be surprised if they started finding out all sorts of cancers are related to cigarettes	Smoker diagnosed with cervical dysplasia
	Social influences from family and friends (n=9)	Cutting back on alcohol, because I would hardly smoke if I didn’t drink. I feel like that’s a trigger for a lot of people	Smoker diagnosed with cervical dysplasia
	Psychosocial stressors/stress (n=5)	Sometimes if I’m really aggravated I’ll have a cigarette	Smoker diagnosed with cervical dysplasia
	Limited ability to access resources for quitting (n=4)	No, I have never been counseled by my healthcare provider. [I received counseling] when I called the New York state hotline. There was like a half an hour…and that’s pretty much the only counseling I got	Former smoker with no history of cervical dysplasia
WHC nurses and office staff (n=10)	Staff lacks resources to offer patients (n=5)	At this particular office we need more resources	Current smoker
	Patient readiness (n=10)	I think when they say, “yeah, yeah I know I need to stop” and then they kind of leave it at that or “I know it’s bad for me, but I don’t want to stop.” It’s kind of hard for me. You can’t make them stop	Non smoker
	Staff ill-equipped to offer tobacco cessation counseling (n=4)	We don’t do any pharmacotherapy but I’ll encourage them to call the New York State Quite line or speak to their primary doctor if they want to go on specific medication	Non smoker

**Table 2:** Recommendations for developing a smoking cessation program, by group.

Group	Themes (# of times participants mentioned them)	Illustrative Quotations	Participant Characteristics
GYN center patients (n=13)	Need for patient-based, individualized approaches to smoking cessation (n=4)	I just think seeing older people who have actually really suffered from smoking. That’s what really kind of got me to quit. It was some real life stories. I clean for a lady who can’t even walk up a half a flight of steps and she has emphysema directly related to smoking and it’s not worth it.	Former smoker with cervical dysplasia
	Address level of readiness for change (n=4)	No, everybody tells you that you’ve got to quit smoking. When I was diagnosed with the cancer was the kicker. That was the reason.	Former smoker, past history of cervical dysplasia and vulvar cancer
	Address social influences on smoking, such as other smokers in the family, friends and partying (n=3)	It was a lot of things…either habit when I was driving, when I was with other friends that smoked or when I was out drinking that I smoked the most.	Former smoker with cervical dysplasia
	Provide assistance and guidance to initiate cessation support in spite of social stresses (n=4)	Half an hour of interview on the phone and a little counseling thrown in there too. They gave like little tips on how to quit smoking, how to cut back on smoking, ideas on how to help you beat the habit.	Former smoker with no history of cervical dysplasia
	Mobile phone not an optimal platform for all patients due to cost, privacy concerns and shared phone plans (n=3)	For me personally, I probably would ignore them [text messages]. I get a lot of messages like that from my phone company and I just delete them because some people are like…oh, I have to pay for text messages…	Smoker with cervical dysplasia
	Automated voice response system not optimal for cessation program due to user fatigue (n=6)	It would probably become annoying after a while.	Former smoker with cervical dysplasia
WHC nurses and office staff (n=10)	Patient readiness (n=10)	I think a patient has to be ready to change. If they are then I think there are a lot of good resources out there for them.	Non smoker
	Personalization to patients’ specific circumstances (n=4)	A great program and I’ve seen it done in a couple of different ways is if you have something where they can get some type of text message reminder alerts that are quick tips. You know, I think that that’s encouraging. I’ve seen it effective for prenatal care.	Current smoker
	Create buy-in from different types of providers to capitalize on different relationships patients have with various office staff (n=3)	A lot of times when a client sees a common person, somebody who’s not up here, but just as equal as they are, they are more receptive to receive it	Non smoker
	Referral to New York State Smoker’s Quit line or primary care physician for cessation support (n=6)	As long as we give them a good link, if they prove they are really ready and they need this…it’s up to the person.	Non smoker
